# Help wanted: developing clinician leaders

**DOI:** 10.1007/s40037-014-0119-y

**Published:** 2014-05-28

**Authors:** James K. Stoller

**Affiliations:** 1 Education Institute – NA22, Cleveland Clinic Foundation, 9500 Euclid Avenue, Cleveland, OH 44195 USA; 2 Jean Wall Bennett Professor of Medicine, Samson Global Leadership Academy Endowed Chair, Cleveland Clinic Lerner College, Cleveland Clinic, Cleveland, OH USA

**Keywords:** Leadership development, Emotional intelligence, Teamwork, Curriculum

## Abstract

Because healthcare faces challenges, such as ensuring quality and access and controlling cost, effective leadership is needed at every level of healthcare organizations. Yet, physicians are trained in clinical and scientific skills but not in leadership competencies. Furthermore, clinicians often feel ill-prepared to assume managerial and leadership roles. To close this gap, training in leadership competencies, such as emotional intelligence, communication, teamwork, and change management, is urgently needed for physicians and clinicians of all disciplines. Leadership training should be multidisciplinary and should begin early in clinicians’ careers.

Healthcare needs highly effective leadership because the challenges that healthcare faces-ensuring quality, providing access, and controlling cost-are formidable and because executing the changes that are needed will continue to be hard. Effective leadership is needed at every level of healthcare organizations-from the bedside to the ward (i.e., in ‘clinical microsystems’ [1]), in the laboratory and procedure suite, to the board rooms that oversee organizations-because pervasive change is needed.

This need for pervasive change frames a paradox in healthcare that requires urgent educational attention and that has important implications for how we train physicians and, indeed, all healthcare providers. Specifically, on the one hand, effective healthcare requires exquisite teamwork by providers. Substantial data support the notion that desirable clinical outcomes-e.g., decreased surgical mortality rates, enhanced diagnostic accuracy, and lower error rates in emergency care [2, 3]-depend on teamwork among caregivers. Furthermore, improving teamwork among providers enhances outcomes. Also, patients clearly judge their care based on the teamwork among caregivers that they perceive. In fact, the perception that ‘the staff worked together to care for me’ is the strongest correlate of a ‘most likely to recommend’ response regarding the healthcare facility. In the era of enhanced awareness of patients’ perceptions, HCAHPS (Hospital Consumer Assessment of Healthcare Providers and Systems, a program for assessing patients’ perceptions of their care tied to reimbursement in the United States Centers for Medicare and Medicaid Services), and of reimbursement that is increasingly tied to patients’ experience, special attention has naturally focused on enhancing teamwork. The teamwork among healthcare providers may also optimally engage patients’ voices.

On the other side of this paradox, despite traits that predispose physicians to crave quality-‘data orientation, relentless pursuit of quality, and a habit of inquiry’ that are perhaps innate [1]-doctors are not trained to lead teams or organizations [4], hospitals are often organized by silos [5], and physicians have traditionally been trained to be ‘lone healers’ [6]. Indeed, it can be said that the methods by which doctors have traditionally been selected and trained conspires against their being followers and therefore blunts their reflexes for collaboration [7]. For example, the emphasis on individual performance that starts with competing for admission to medical school and continues throughout training to initial certification to practice medicine and then to the required periodic recertification fosters an individual focus. Also, the long and hierarchical nature of medical training cultivates a sense of having ‘arrived’ on completion of training that can undermine valuing others’ input. The risk of ‘extrapolated authority,’ i.e., extending the sense of authority that comes from clinical mastery to contexts for which clinical mastery does not matter-like waiting in line at a restaurant or driving on a highway-also undermines physicians’ ‘followership.’ Finally, clinical training that appropriately indicates a focus on the patient’s presenting problem and on underlying ‘lesions’ naturally creates a deficit-based focus that hampers clinicians’ abilities to think appreciatively or, in systems contexts, to see the full range of organizational possibility [7]. The rigor of clinical training and the time-honored approach to clinical problem-solving causes clinicians to focus on organizational problems only rather than on organizational possibilities. Yet, advocates of ‘appreciative inquiry’ [8] argue that fully realizing and unleashing the systemic possibilities that are so critically needed to effect healthcare reform and to change from ‘volume-based’ to ‘value-based’ care requires an appreciative perspective.

The gap between the need for leadership in healthcare and leadership preparation during medical training has been amply recognized both by medical trainees and by scholars of organizational behaviour. For example, a survey of surgical trainees [9] indicated that although 92 % of respondents rated 18 leadership competencies as somewhat or very important (e.g., conflict resolution, negotiation, time management, and leadership training), at least 50 % of trainees rated themselves as either not competent or minimally competent in most, and 96 % and 78 %, respectively, rated themselves as not or minimally competent in conflict resolution and leadership theory - arguably especially critical competencies that are needed to lead. As long ago as 1978 in a paper entitled ‘Why hasn’t organizational development worked (so far) in medical centres?,’ Weisbord [5] pointed out that ‘Science-based professional work differs markedly from product-based work. Health professionals learn rigorous scientific discipline as the ‘content’ of their training. The ‘process’ inculcates a value for autonomous decision-making, personal achievement, and the importance of improving their *own* performance, rather than that of any institution.’ Westerman [4] reported that newly appointed consultants who have just completed their medical training perceived themselves to be well-trained regarding medical and clinical skills, but unprepared regarding ‘generic competencies such as supervision, leadership, management, and handling financial issues.’ Other recent observers have called for training physicians in leadership competencies such as teamwork, communication, systems thinking, and emotional intelligence. That emotional intelligence matters for leadership success in healthcare has been reported by several studies. In discussing correlates of success and failure among ten chairs of academic internal medicine departments, Lobas [10] noted that ‘Emotional intelligence and its concomitant skills are the most essential competencies for leaders to succeed in academic institutions. The ten chairs emphatically stated that this ability was fundamental to their success and its absence the cause of their failures. They suggested that the absence of emotional intelligence often resulted in the demise of chairs and contributed to the high turnover among colleagues.’ Furthermore, Lobas recommended that emotional intelligence should be assessed as a key component of leadership selection. Similarly, in an email survey of chairs of academic psychiatry departments, Keith and Buckley [11] identified emotional intelligence competencies such as interpersonal communication, integrity and honesty, altruism, and tolerance as essential.

The gap between the need for leadership and clinicians’ lack of preparedness to lead provides a clarion call for a significant, broad-based response. A successful response must address all communities of caregivers and must do so strategically throughout all phases of their training and practice. To optimize learning, the training should be undertaken in an iterative fashion over the course of training and beyond and should be informed by the principles of a ‘spiral curriculum’ [12]. Specifically, teaching leadership development should focus on the elements of leadership and of emotional intelligence-self-awareness, self-management, social awareness, and managing relations with others and the specific component competencies-that are most relevant to the learner at each phase of training. For example, a medical school leadership curriculum should address medical students’ keener interest in mastering time management, achievement, and emotional self-control, while a leadership curriculum for chief residents and junior faculty might focus more on negotiation, conflict resolution, change management, and teambuilding competencies.

For physicians, attention to leadership skills should begin during selection for medical school admission and leadership and emotional intelligence training must suffuse undergraduate medical education. Furthermore, undergraduate medical curricula should be designed in ways that foster teamwork and lifelong learning while discouraging the dysfunctional interpersonal competition that may also underlie the ‘lone healer’ [6] nature of physicians. Approaches such as problem-based learning, team-based learning, and portfolio-based assessment align with these goals. Selection for graduate medical education (GME) positions should include attention to leadership competencies and GME curricula should extend leadership training through an emphasis on teamwork, collaboration, and emotional intelligence. Indeed, teamwork and leadership competencies are currently emphasized in some undergraduate [13] and graduate medical curricula [14]; for example, the Royal College of Physicians and Surgeons of Canada has endorsed the CanMEDS model, which cites six key competencies for training, of which expertise in management is one. Success as a manager includes the ability to ‘serve in administrative and leadership roles, as appropriate,’ and to ‘lead or implement a change’ [14]. Finally, criteria for selecting faculty for clinical and scientific leadership positions in healthcare organizations must go beyond the traditional ‘threshold’ criteria of academic performance, grantsmanship, and fundraising prowess and should focus on leadership competencies [5–7, 10]. Recognizing that success in faculty roles requires leadership skills, UCLPartners, an academic health science partnership in London, has implemented a formal leadership curriculum for selected National Health Service faculty [15]. Many academic medical centres and professional societies have also implemented leadership development curricula [16, 17].

The need to train leaders is by no means limited to physicians. Nurses, physicians’ assistants, and allied health providers of all types must also hone their competencies to lead, to follow, and to be emotionally intelligent clinicians. Their respective curricula must also address these leadership competencies in an iterative and progressive way. Indeed, this diffuse need for leadership development underscores the need and opportunity for interprofessional education with physicians, nurses, and allied health professionals at times learning leadership skills together in intact teams. As an example, Neily et al. [3] showed that teamwork training for multidisciplinary surgical teams using a curriculum based on crew resource management was associated with decreased surgical mortality rates.

These considerations about the emerging climate of healthcare, the need for highly effective leadership, and the gap between clinicians’ training and the need for leadership frame a triple call to action. First, urgent attention is needed to train clinicians in leadership and emotional intelligence competencies. Second, this training must be broadly available to all healthcare providers and interprofessional education should be implemented where possible and appropriate. Third, attention to leadership competencies should inform selection of individuals for these professions and such training should begin early in their professional curricula and proceed iteratively and progressively throughout training and into their early careers. A ‘Help Wanted’ sign is flashing for healthcare. Let’s fill the positions.

## References

[1] Bohmer RMJ (2013). Leading clinicians and clinicians leading. New Engl J Med.

[2] Wheeler D, Stoller JK (2011). Teamwork, teambuilding and leadership in respiratory and healthcare. Can J Resp Ther.

[3] Neily J, Mills PD, Young-Xu Y (2010). Association between implementation of a medical team training program and surgical mortality. JAMA.

[4] Westerman M. Mind the gap: the transition to hospital consultant. Perspect Med Educ 2013. doi 10.1007/s40037-013-0104-x. 10.1007/s40037-013-0104-x PMC4078059 24327050

[5] Weisbord MR (1976). Why organization development hasn’t worked (so far) in medical centers. Health Care Manag Rev.

[6] Lee TH. Turning doctors into leaders. Harv. Bus Rev. April 2010;88(4):50–58. 20402055

[7] Stoller JK (2004). Can physicians collaborate? A review of organizational development in healthcare. OD Pract.

[8] May N, Becker D, Frankel R, et al. Appreciative inquiry in Healthcare Crown Custom Publishing, 2011.

[9] Itani KMF, Liscum K, Brunicardi C (2004). Physician leadership is a new mandate in surgical training. Am J Surg.

[10] Lobas JG (2006). Leadership in academic medicine: capabilities and conditions for organizational success. Am J Med.

[11] Keith SJ, Buckley PF (2011). Leadership experiences and characteristics of chairs of academic departments of psychiatry. Acad Psychiat.

[12] Taylor C, Farver C, Stoller JK (2011). Perspective: can emotional intelligence training serve as an alternative to teaching professionalism?. Acad Med.

[13] Dannefer E, Henson L (2007). The portfolio approach to competency-based assessment at the Cleveland Clinic Lerner College of Medicine. Acad Med.

[14] Royal College of Physicians and Surgeons of Canada. CanMEDS. www.royalcollege.ca. Accessed 22 Dec 2013.

[15] UCLPartners. www.uclpartners.com. Accessed 23 Dec 2013.

[16] Stoller JK (2009). Developing physician-leaders: need and rationale. J Health Admin Ed.

[17] Stoller JK, Berkowitz E, Bailin P (2007). Physician management and leadership education at the Cleveland Clinic Foundation: program impact and experience over 14 years. J Med Pract Manag.

